# Multi-color live-cell super-resolution volume imaging with multi-angle interference microscopy

**DOI:** 10.1038/s41467-018-07244-4

**Published:** 2018-11-16

**Authors:** Youhua Chen, Wenjie Liu, Zhimin Zhang, Cheng Zheng, Yujia Huang, Ruizhi Cao, Dazhao Zhu, Liang Xu, Meng Zhang, Yu-Hui Zhang, Jiannan Fan, Luhong Jin, Yingke Xu, Cuifang Kuang, Xu Liu

**Affiliations:** 1 State Key Laboratory of Modern Optical Instrumentation, College of Optical Science and Engineering, Zhejiang University, Hangzhou, Zhejiang, 310027 China; 2 Shanxi Provincial Key Laboratory for Biomedical Imaging and Big Data, North University of China, Taiyuan, Shanxi 030051 China; 3 Britton Chance Center for Biomedical Photonics, Wuhan National Laboratory for Optoelectronics-Huazhong University of Science and Technology, Wuhan, Hubei 430074 China; 4 Key Laboratory for Biomedical Engineering of Ministry of Education, Department of Biomedical Engineering, Zhejiang University, Hangzhou, 310027 China; 5 Collaborative Innovation Center of Extreme Optics, Shanxi University, Taiyuan, Shanxi 030006 China

## Abstract

Imaging and tracking of near-surface three-dimensional volumetric nanoscale dynamic processes of live cells remains a challenging problem. In this paper, we propose a multi-color live-cell near-surface-volume super-resolution microscopy method that combines total internal reflection fluorescence structured illumination microscopy with multi-angle evanescent light illumination. We demonstrate that our approach of multi-angle interference microscopy is perfectly adapted to studying subcellular dynamics of mitochondria and microtubule architectures during cell migration.

## Abstract (web summary)

3D super-resolution imaging of dynamic processes in live cells is still challenging, especially in a large field of view. Here the authors combine SIM with multi-angle evanescent light illumination and achieve improved lateral and axial resolution, with stack acquisition time in the range of 1–2 s.

## Introduction

Observing three-dimensional (3D) volumetric subcellular structures and functions is essential for biological research. For this reason, a variety of powerful super-resolution fluorescence imaging techniques have been proposed. One approach for overcoming the diffraction limit in lateral dimension is activating and localizing single molecule^1–7^. Techniques like modulating the point spread function (PSF)^1–4^, increasing the numerical aperture (NA) of the system^4–6^, or detecting supercritical light^7^ can improve the axial resolution. Similarly, 3D nanometer details can be resolved by combining stimulated emission depletion microscopy (STED) with an NA increasing technique^8^.

However, although these 3D super-resolution approaches are appropriate to the study of relatively static structures, such as stationary microtubules and nuclear pore complexes, they are difficult to use for imaging and tracking the real-time evolution of live cells in a large field of view because of the requirements of high excitation intensities of 10^3^–10^8^ W cm^−2^ (far beyond the safe intensity of 0.1 W cm^−2^ for live-cell imaging), specialized fluorophores and labeling strategies, and a long imaging capturing process.

The aforementioned shortcomings are overcome by 3D structured illumination microscopy (3D SIM)^9–11^, which can image 3D living samples labeled by conventional dyes with an excitation intensity of 1–10 W cm^−2^. However, the limited spatial resolution, which is typically improved twofold or less in lateral and axial resolutions, limits its biological applications^12^.

The evanescent wave is widely used in optical microscopy to illuminate near-surface cell structures on an axial scale down to a few hundred nanometers. By leveraging the exponential decay of the evanescent field from the coverslip, microscopy based on total internal reflection fluorescence (TIRF) can further improve the axial resolution by recording a series of images at different incident angles^13^ or different wavelengths^14^. Nevertheless, the immense difference between the wide-field lateral resolution and 10–50-nm axial resolution of TIRF engenders its poor performance in volumetric super-resolution imaging.

Inspired by these existing challenges, we designed and implemented a technique for 3D multi-color live-cell super-resolution volume imaging by incorporating SIM with multi-angle evanescent light illumination. We refer to the proposed approach as multi-angle interference microscopy (MAIM) (Supplementary Figs. 1–3). The introduction of evanescent wave improves the signal-to-noise ratio (SNR) and optical sectioning capacity of the structured illumination methodology (Supplementary Fig. 4). Compared to other 3D super-resolution techniques^1–8^, MAIM has an advantage with respect to temporal resolution because of the smaller number of required raw images. Moreover, a lower requirement for the surface energy density of the illumination light, which is even lower than that of 3D SIM, enables its use for probing specific long-term dynamics in living cells without photodamage or photobleaching. Furthermore, the portability of the sample preparation protocols and experimental implementation allows it to be easily adapted to a wide range of biological studies.

## Results

### MAIM theory and demonstration

**Fig. 1:**
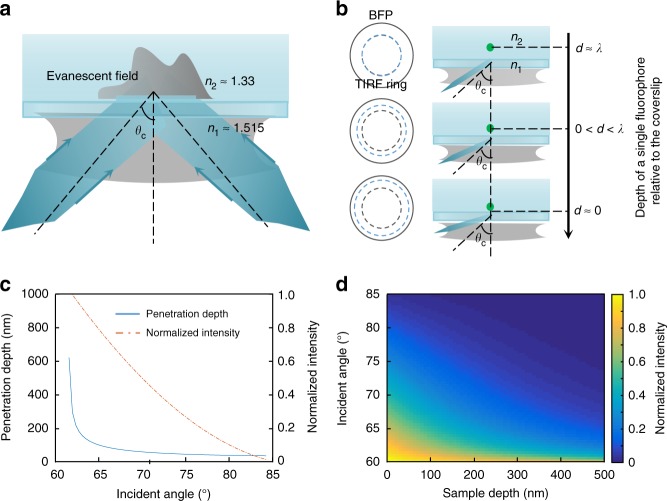
MAIM theory. **a** Two opposite evanescent waves are generated when two light beams are incident from both sides of the optical axis at an angle beyond the critical angle *θ*_c_, which can interfere with each other to modulate the sample frequencies. **b** The position of the light focus at the back focal plane (BFP) of the objective determines whether or not the TIRF will appear. The larger the angle of incidence is, the closer the focal point is to the edge of the BFP and the shallower the penetration depth of the evanescent wave is. By changing the incident angle, we can obtain a series of images at different depths of the sample. **c** Simulated relationships between the incident angle and the penetration depth and normalized intensity of the evanescent wave at *z* = 0. **d** Simulated intensity distribution of the evanescent wave as a function of the incident angle and the sample depth

In MAIM, we used an established TIRF-SIM algorithm to achieve lateral super-resolution^15^. Following the robust procedure for breaking the lateral optical diffraction limit by down-shifting high-frequency components, we first recorded nine TIRF-SIM raw images (Fig. 1a). To enhance the axial resolution, we collected a set of multi-angle TIRF (MA-TIRF) images by azimuthal averaged over different evanescent wave propagation directions (so-called “MA-Ring-TIRF”), which contain different depth information of the sample (Fig. 1b–d, Supplementary Fig. 5). The lateral TIRF-SIM super-resolution image is then thresholded, and serves as a binary spatial mask to subtract the local background, abandon the diffraction-limited information, and segment the interesting sample information from the MA-Ring-TIRF raw images. The resulting segmentation image stack is taken as the input of the 3D super-resolution reconstruction. By treating them as a convex optimization problem, a precise knowledge of our system parameters, such as the quantum efficiency, absorption rate, detection efficiency, and PSF, was not required. We then solved the problem with our previously reported fast and efficient reconstruction algorithm based on distributed optimization acceleration^16^ (Supplementary Fig. 3). With these procedures, MAIM enabled us to map the surface morphology with a sub-100-nm lateral resolution and axial architectures with an approximately 40-nm axial resolution over a 600-nm depth volume. The typical acquisition time of one volume stack in MAIM is 1–2 s. We demonstrated this by tracking the fast temporal evolution of Atto 647N-labeled mitochondria and SiR-tubulin-labeled microtubules in live U2OS cells.

**Fig. 2:**
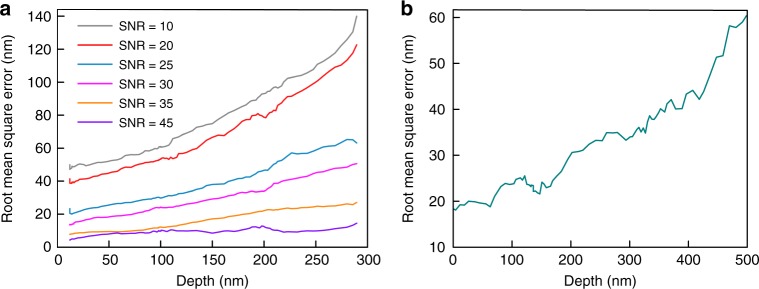
Axial localization accuracy of MAIM. **a** Theoretical root mean square error of the calculated depth from the true position under different depths and different SNRs. **b** Experimental root mean square error of the calculated depth from the true position under different depths with the silica sphere ground-truth sample

We analyzed the parameters affecting the reconstruction by conducting simulations of 3D microtubule structures incorporating Gaussian white noise while changing the axial position, incident angle range, and angle number. Supplementary Fig. 6a, b present the 3D ground-truth data and the reconstructed depth data (SNR = 30, 20 angles from 61.5° to 71°). We found that the root mean square error of the calculated depth from the true position heavily depends on the SNR, and can be below 5 nm when a fluorescent emitter is bright and near the coverslip (Fig. 2a). Further, to reduce the influence of the refractive index mismatch on the reconstruction (Supplementary Fig. 6e), we experimentally determined the refractive index of each sample before the reconstruction (Supplementary Note 1). Other reconstruction parameters, for example, the angular steps and incident angle varying range, have relatively less effects on the reconstruction (Supplementary Fig. 6c, d). From the analysis, a typical 10–20 angle number from 61.5° to 71° is the optimal choice for MAIM reconstruction to provide the highest spatial and temporal resolutions. Furthermore, we used gradually spaced lines (Argo-HM) and silica microsphere (Bangs Laboratories, Inc.) ground-truth samples to experimentally validate MAIM (Fig. 2b and Supplementary Fig. 7). The lateral resolution enhancement was confirmed by separating 100-nm distance in the spaced line sample (Supplementary Fig 7a–c). The measured z-positions of the spheres distributed well around the theoretical z-profile. The final fitted averaged sphere diameter of 4.98 μm was slightly larger than the theoretical value of 4.86 μm possibly reflecting the physical uniformity of the spheres (±0.47 μm) (Supplementary Fig. 7d–f). The experimental axial root mean square error had the same descending trend with the simulation result when the depth increases, and was ~20–60 nm in the depth range from 0 to 500 nm (Fig. 2b).

### Fixed cell imaging

**Fig. 3:**
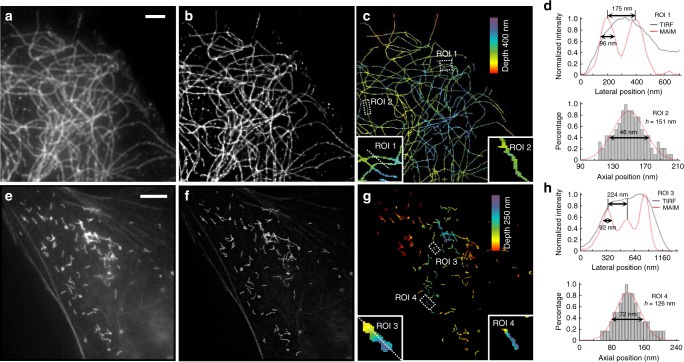
Multi-angle interference imaging of microtubules and peroxisomes (PMP70). **a** TIRF image, **b** lateral super-resolution image, and **c** 3D volume super-resolution image of microtubules labeled with Alexa Fluor 488 (*λ* = 488 nm) in U373 cells. The regions of interest (ROI) are boxed in white, and their magnified images are shown at the bottom-left and bottom-right of **c**. **d** The plot (top) and the histogram (bottom) show the lateral profile of the white line in ROI 1 and the axial profile of ROI 2, respectively. The mean depth (*h*) above the coverslip is calculated over a range of 90–210 nm for ROI 2. **e** TIRF image, **f** lateral super-resolution image, and **g** 3D volume super-resolution image of peroxisomes (PMP70) labeled with STAR 580 (*λ* = 561 nm) in U2OS cells. The ROIs are boxed in white, and their magnified images are shown at the bottom-left and bottom-right of **g**. **h** The plot (top) and the histogram (bottom) show the lateral profile of the white line in ROI 3 and the axial profile of ROI 4, respectively. The *h* above the coverslip is calculated over a range of 0–240 nm for ROI 4. Note that the removed filamentous structures in **g** compared with **e**, **f** are the cross-color filaments labeled with another kind of fluorescent dye. Scale bars, 3 μm (**a**–**c**), and 5 μm (**e**–**g**)

To demonstrate the performance of MAIM in cell imaging, we conducted a series of experiments utilizing our MAIM setup (Supplementary Figs. 1 and 2). Firstly, we imaged the microtubule networks labeled with Alexa Fluor 488 in U373 cells grown in a two-well glass chamber (Fig. 3a–d). After immunofluorescence staining, the cell membrane became permeable for antibodies to enter; therefore, the refractive index inside the cell could be matched with the surrounding aqueous buffer solution. A blue laser (*λ* = 488 nm) was used to excite fluorophore under the TIRF configuration. The excitation power was maintained constant throughout the whole imaging process (0.89 W cm^−2^). Multi-azimuthal-angle and multi-phase raw images were acquired to reconstruct the lateral super-resolution image of the sample (Fig. 3b). The sub-diffraction structures in the TIRF image (Fig. 3a) were now clearly resolved.

By acquiring an image stack of 20 incident angles with a 0.50° angular step size starting from 61.64°, we reconstructed the 3D volume distribution of the microtubules with the lateral and axial resolutions of 98 and 46 nm, respectively, up to approximately 400 nm in depth (Fig. 3c, d). The color-coded depth visualization revealed a continuous upward bending of the microtubule network from the leading edge toward the center. Six different algorithms for reconstructing the multi-angle interference image stack were compared on this image series (Supplementary Fig. 8). A visual reconstruction of peroxisomes (PMP70) labeled with STAR 580 (*λ* = 561 nm) at 20 incident angles with a 0.50° angular step size starting from approximately 61.91° showed fine 3D details, revealing distinct topographical features (Fig. 3e–h). The lateral resolving ability of MAIM was confirmed by discriminating the circular shape of the peroxisomes (Fig. 3f, g), which was not straightforward in the conventional TIRF image and multi-angle total internal reflection fluorescence (MA-TIRF) reconstructed image (Fig. 3e and Supplementary Fig. 9). The histogram of several peroxisomes showed a 72-nm axial resolution and a 126-nm mean depth (Fig. 3h).

**Fig. 4:**
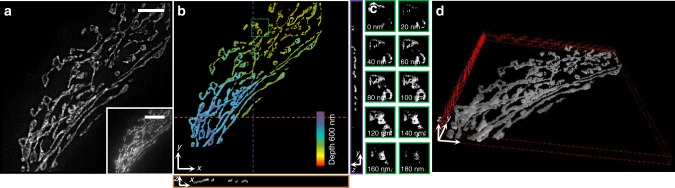
Multi-angle interference imaging of mitochondria. **a** Lateral super-resolution image and **b** 3D volume super-resolution image of mitochondria. The bottom-right of **a** shows the corresponding TIRF image. The bottom and right images of **b** show the corresponding vertical cross-section along the respective orange and purple dotted lines in **b** produced using ImageJ3D. **c** Consecutive x**–**y sections of the green boxed region in **b** from a 0 to 180-nm depth, showing the hollow shape and morphology change of individual mitochondria. **d** Three-dimensional (3D) volume visualization of the mitochondria using ImageJ3D. The size of the reconstructed 3D region is 32 × 32 × 0.6 μm^3^. Scale bars, 5 μm (**a**, **b**), and 10 μm (bottom-right of **a**)

We next applied MAIM to image the mitochondrial network in fixed bovine pulmonary artery endothelial cells labeled with MitoTracker® Red CMXRos (Cat. no. M7512) (*λ* = 561 nm) at the illumination power of 1.32 W cm^−2^ (Fig. 4). The MAIM image reconstructed from one volume of 30 incident angles with a 0.33° angular step size starting from approximately 61.51° showed more significantly improved lateral and axial resolutions over the conventional diffraction-limited TIRF image. We observed three distinct forms of mitochondrial morphology: many interconnected tubular networks, intermediate mitochondrial structures, and a few dispersed globular structures, which likely indicated the interphase stage of the cell^17^ (Fig. 4b). A series of 3D super-resolution images revealed the hollow shape and morphology change of individual mitochondrial outer-membranes spanning multiple axial slices from a depth of 0 nm to 180 nm relative to the coverslip (Fig. 4c). The change of the whole imaging area was also given (Supplementary Fig. 10). Such process of continuous change in the mitochondrial hollow structure was also previously reported using 3D STED^8^ and 3D STORM^18^. The reconstructed images at different depths given by MAIM were converted into a 3D near-surface volume view of 32 × 32 × 0.6 μm^3^, which more distinctly showed the 3D morphology change of the mitochondria (Fig. 4d and Supplementary Movie 1).

### Multi-color imaging

**Fig. 5:**
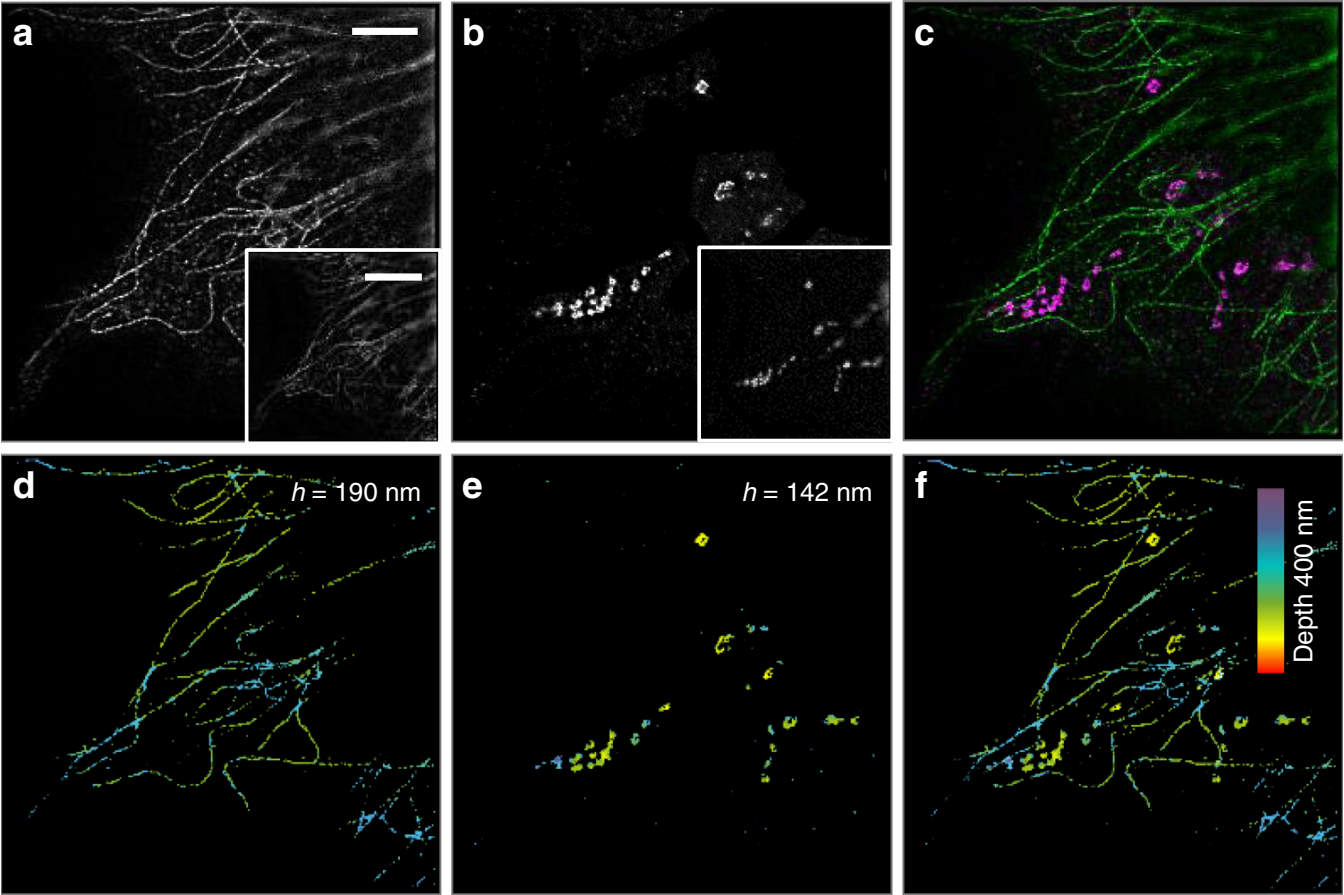
Multi-color multi-angle interference imaging of microtubules and mitochondria. **a** Lateral super-resolution image of microtubules stained with Alexa Fluor 488 (*λ* = 488 nm) and **b** outer mitochondrial membrane 20 (TOM20) stained with Alexa Fluor 568 (*λ* = 561 nm) in the same U373 cells. The bottom-right of **a** and **b** shows the corresponding TIRF images. **c** Dual-color merging lateral super-resolution co-localization image of microtubules (green) and mitochondria (magenta). **d** Three-dimensional (3D) volume super-resolution image of the same microtubules and **e** mitochondria. The *h* of the microtubules and mitochondria is 190 and 142 nm, respectively. **f** Merging super-resolution volume co-localization image of microtubules and mitochondria. Scale bars, 4 μm (**a**–**f**), and 8 μm (bottom-right of **a** and **b**)

At this point, we simultaneously measured the 3D distributions of microtubules stained with Alexa Fluor 488 (*λ* = 488 nm) and mitochondria stained with Alexa Fluor 568 (*λ* = 561 nm) in the same U373 cell (Fig. 5). The raw image stacks were all acquired at 16 different incident angles with a 0.50° angular step size starting from 61.64°. As an organelle providing energy to cells, mitochondria generally distribute around the nucleus and near the microtubules, which was in accordance with our co-localization imaging result (Fig. 5c, f). At the axial direction, we quantitatively observed that microtubules were slightly deeper than mitochondria (Fig. 5d–f), verifying the capability of MAIM for multi-color super-resolution volume imaging and co-localization of different cell components.

### Live-cell imaging

**Fig. 6:**
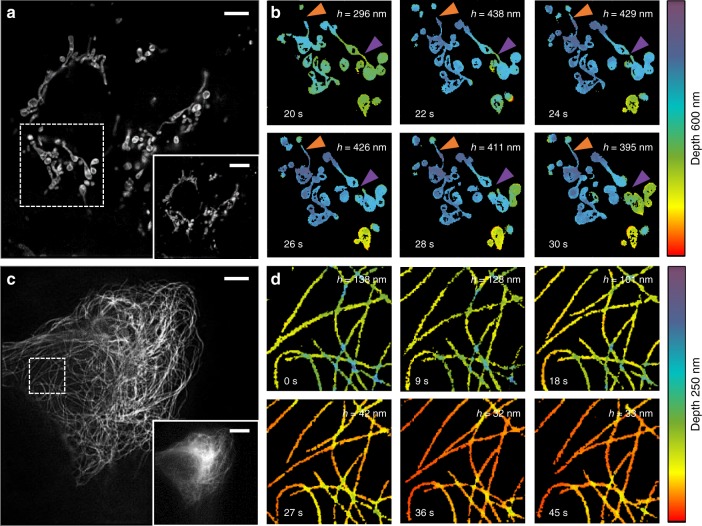
Live-cell time-lapse multi-angle interference imaging of mitochondria and microtubules. **a** Lateral super-resolution image of mitochondria in live U2OS cells labeled with Atto 647N (*λ* = 640 nm). The bottom-right shows the corresponding TIRF image. **b** Time-lapse 3D volume super-resolution image sequence of the ROI marked with a white box in **a**, showing the changes of the lateral and axial positions and the fission (purple triangle) and fusion (orange triangle) of mitochondria. The *h* varies between 296 nm and 438 nm during the interval of 20 s to 30 s. **c** Lateral super-resolution image of microtubules in live U2OS cells labeled with SiR-tubulin (*λ* = 640 nm). The bottom-right shows the corresponding TIRF image. **d** A time-lapse 3D volume super-resolution image sequence of the ROI marked with a white box in **c**, which shows the changes of lateral and axial positions of microtubules. The *h* changes from 138 nm to 32 nm during the interval of 0 s to 45 s. Scale bars, 10 μm (**a, d**), and 5 μm (bottom-right of **a** and **b**)

The fact that MAIM is a powerful tool to study the high dynamics of intracellular architectures was demonstrated by imaging mitochondria and microtubules in different live U2OS cells labeled with Atto 647N (*λ* = 639 nm) and SiR-tubulin (*λ* = 639 nm), respectively. Atto 647N has been increasingly used as the optimal labeled dye in live-cell super-resolution imaging because of its high luminous efficiency and excellent photostability^19^. After staining with Atto 647N, U2OS live cells were imaged using our method. The result showed that the mitochondria architecture within a 600-nm depth was reconstructed from a 3D volume corresponding to ten incident angles with a 0.67° angular step size starting from 61.51° (Fig. 6a,b and Supplementary Movie 2 and 3).

A time-lapse sequence of multi-angle interference image stacks of live cells was acquired at a temporal resolution of approximately 1 s per volume with an exposure time of 50 ms and an excitation power of 0.93 W cm^−2^. Few instant color-coded super-resolution images showed the axial depth change (296**–**438 nm) and lateral evolution of individual mitochondrion and multiple mitochondria, such as migration, retraction, fusion, and fission (Fig. 6b). As visualized at the connection of moving mitochondria, the fission appeared concomitant to constriction/protrusion events (Fig. 6b, purple arrowheads). After fission, we could observe the downward movement of the forming mitochondria because of the high axial resolving capacity of our approach. The 3D dynamics and volume rendering of mitochondrial membranes in the image sequence were both consistent with similar data from the COS-7 cell^20^.

Considering the relatively slow dynamic behavior of the microtubules compared with the mitochondria in our experiments, their image series was recorded with a 100-ms exposure time and a 0.66Wcm^−2^ illumination power at one volume of 20 incident angles with a 0.50° angular step size starting from approximately 61.91° corresponding to 250 nm in the depth of the cells (Fig. 6c, d). From the color changes of the reconstructed super-resolution volume images, we observed that their depth tended to be downward (Fig. 6d). In a more quantitative measurement, the average depth of the selected microtubules varied from 138 nm to 32 nm during the 45-s recording time. The lateral real-time movements of extension, contraction, and waving of the network were also tracked (Fig. 6d).

## Discussion

In summary, we demonstrated herein that MAIM is a powerful technique that provides a combination of super-resolution volume imaging, live-cell imaging, multi-color imaging, a long time series, and low photodamage, which other 3D imaging techniques have difficulty providing. The proposed technique achieves this combination without requiring specially-prepared biological samples. Consequently, it is very suitable for the investigation of dynamic molecular behaviors that are proximal to the cell membrane, and can be widely applicable to some critical issues, such as vesicle endocytosis and exocytosis, membrane transport, membrane heterogeneity, and mechanobiology. With respect to the prominent features of our method, it can provide absolute axial sectioning and depth positioning of fluorescent structures relative to the coverslip (Fig. 4 and Supplementary Fig. 10).

Moreover, in real-time live-cell imaging, the MAIM imaging speed depends only on the camera image acquisition time. Theoretically, its speed can reach up to approximately 100 Hz, which means the temporal resolution of our MAIM system can be 3**–**5 volume per second. However, in practice, the local concentration, quantum efficiency, photostability and brightness of dyes, as well as the laser excitation power, field of view size, and system detection efficiency must be comprehensively considered when recording images. With the rapid development of faster cameras, brighter and more stable fluorescent dyes, alternative cell-permeable organic fluorescent probes, and more mature labeling strategies, the simultaneous multi-color super-resolution imaging of different, but correlative dynamic components in a living system is possible to analyze the interactions among these components. This is difficult to achieve otherwise with other single molecule localization-based techniques.

## Methods

### Multi-angle interference microscopy instrumentation

All optical elements used in our technique were mounted on a 750 × 750 × 60 mm optical breadboard (Thorlabs, B7575L) to minimize external mechanical vibrations. Three lasers were equipped for fluorescence excitation: a 300-mW, 488-nm laser (Coherent, Sapphire 488-300 CW CDRH); a 300-mW, 561-nm laser (Coherent, Sapphire 561-300 CW CDRH); and a 1-W, 639-nm laser (Coherent, Genesis MX 639–1000). The illumination wavelength and the intensity of the lasers were controlled by an acousto-optical tunable filter (AOTF) (AA Quanta Tech, AOTFnC-400.650-TN). After the AOTF, beams were coupled into a single mode polarization maintaining fiber (Oz Optics, core size of 3 μm, NA 0.11), then collimated by a beam collimator (Thorlabs, ZC618FC). Parallel beams were separated into two paths with orthogonal polarization directions by a polarization beam splitter (PBS) (Thorlabs, CCM1-PBS251/M). Before that point, the intensity ratio of two paths was controlled by a half-wave plate (Thorlabs, AQWP05M-600) to ensure the high modulation contrast of the interference pattern. The two light beams were then directed into two construction-symmetric paths, which consisted of two reflecting mirrors to alter the propagation direction of the beam, a galvanometer set (Cambridge Technology, CTI 8310k) that serves to change the incident angle and rotate the orientation of the interference pattern, and a scanning lens (Thorlabs, SL50-CLS2) to rectify and focus the light. The difference was that a half-wave plate (Thorlabs, AQWP05M-600) and a piezoelectric stage (PI, 753.1, 5 kHz) were inserted into one path to adjust the polarization and shift the interference pattern phase. Two focusing beams were combined by a beam splitter (BS) (Thorlabs, CCM1-BS013) and reimaged in opposite form near the edges of the BFP of a high-NA oil-immersion objective (Nikon, 100 × /1.49 TIRF) by a 4f configuration constructed from two tube lenses (Thorlabs, TTL200-A, *f* = 200 mm) and a periscope. A polarization rotator was placed behind the BS consisting of a liquid crystal cell (LC, Meadowlark, SWIFT) and an achromatic quarter-wave plate (Thorlabs, SAQWP05M-700) to simultaneously rotate the polarization of the two focused beams to s-polarization. After entering the TIRF input port of an inversed microscope (Nikon, Ellipse Ti) equipped with input and output side ports and an automated XY translation stage, the beams sequentially passed through a dichroic mirror cube (DM) (Chroma, C174298) and the objective. The fluorescence emitted by the sample was collected by the objective, reflected with the DM, focused by a *f* = 200 mm tube lens that was internal to the microscope and detected with an electron-multiplying charged-coupled device (EMCCD) camera (Andor, iXon Ultra 888) mounted on the microscope output side port. Supplementary Figs. 1 and 2 present all the elements described earlier. The relationship between the galvanometer voltage and the incident angle was calibrated by measuring light positions and imaging the objective BFP (Supplementary Fig. 11, Supplementary Note 1, and Supplementary Movie 4).

The total magnification between the sample and EMCCD was 250, leading to an imaging pixel size of 52 nm. Although somewhat oversampled, the raw images were binned from 1024 × 1024 to 512 × 512 to meet the Nyquist sampling limit and reduce the acquisition time. The experimental laser power varied from 0.66 W cm^−2^ to 1.32 W cm^−2^ with reliance on the particular sample and exposure time. Power measurements were performed at the output of the high-NA objective lens when light was incident normally by the microscope silicon photodiode power probe (Thorlabs, S170C). The typical exposure time of EMCCD was 50**–**100 ms with the electron-multiplying gain of 150**–**300, resulting in a 1**–**2-s acquisition rate per 3D near-surface volume for a field of view of 512 × 512 pixels. The time consideration among the illumination, exposure, and acquisition caused less photodamage and photobleaching while engendering an optimal SNR and temporal resolution for the given light dose to the sample.

For the multi-color imaging, the MAIM system did not need to correct the chromatic aberration that a spatial light modulator (SLM)-based SIM must consider because of its constraints on pattern generation resulting from the discrete and finite pixel arrangement and the wavelength-dependent nature of SLM^11^.

### Fixed sample preparation

For the fluorescent nanoparticle preparation used in Supplementary Fig. 11, F8803 particles were diluted in absolute ethyl alcohol with the dilution ratio of 1:1000, then vibrated by an ultrasonic washer for 20 min. The coverslips were coated with poly-l-lysine (Sigma) for 10 min. Subsequently, the particle solution was incubated on coverslips. After naturally spreading, the coverslips were all mounted on microscope slides with the Mowiol mounting medium. For the fixed cell preparation used in Figs 3a–d, 5, and Supplementary Figs. 4,5,8, U373 cells (ATCC) were cultured in a 100-mm culture dish (Corning) with Dulbecco’s Modified Eagle’s Medium (DMEM; Corning) supplied with 10% fetal bovine serum (FBS) (Gibco). The culture dishes were retained in the incubator at 37 °C with 5% CO_2_. Cells were seeded in 35-mm glass-bottom dishes (Cellvis) and fixed and permeabilized with a **–**20 °C methanol for 10 min. The fixed cells were rinsed thrice with phosphate buffered saline and blocked with a blocking buffer containing 3% BSA and 1% goat serum for 1 h. Tubulin antibody (Abcam, ab7751), and Goat anti-Mouse IgG (H + L) Cross-Adsorbed Secondary Antibody, Alexa Fluor 488 (Invitrogen, A-11001) were diluted at 1:200 in the blocking buffer. The cells were incubated with primary antibody overnight at 4 °C. After washing thrice with phosphate buffered saline, a secondary antibody incubation was conducted at room temperature for 1.5 h. The sample was rinsed thrice with phosphate-buffered saline and imaged with the desired imaging system. For the multi-color sample preparation used in Fig. 5, Tom20 antibody (Santa cruz, sc-11415) and Goat anti-Mouse IgG (H + L) Cross-Adsorbed Secondary Antibody, Alexa Fluor 568 (Invitrogen, A-11004) were used to label the mitochondria of the U373 cells. The silica microspheres (Bangs Laboratories, Inc., *n* = 1.42, diameter = 4.86 ± 0.47 μm) used in Supplementary Fig. 7d–f were labeled with Alexa Fluor 488 dye, immersed in index-matched medium (70% glycerol/water solution) and attached to the coverslip surface. The peroxisomes (PMP70) stained with STAR 580 used for biological examinations (Fig. 3e–h) were commercial samples from Abberior Instruments. The gradually spaced line ground-truth sample used in Supplementary Fig. 7a–c was commercialized from Argolight.

### Cell culture and live-cell sample preparation

For the live-cell sample preparation used in Fig. 6, U2OS cells were purchased from Boster Biological Technology Co., Ltd., Wuhan, China, and grown at 37 °C in 5% CO_2_ in McCoy’s 5 A (Modified) medium (GIBCO) supplemented with 10% FBS (Gibco). The cells were seeded on a glass-bottom dish (Φ 12 mm, Thermo Fisher Scientific, Inc.). After an overnight incubation, the cells were washed thrice with phosphate-buffered saline.

For the microtubule labeling, the cells were incubated with 1-μM SiR-Tubulin (New England BioLabs) for 1 h. From that point, the supernatant was discarded, and the cells were washed twice gently with phosphate-buffered saline and incubated in McCoy’s 5A (Modified) medium containing 10% FBS for 1 h. Before imaging, the medium was changed to phenol red-free DMEM (Thermo Fisher Scientific, Inc.).

For labeling mitochondria with Atto 647N in live U2OS cells, Atto 647N (25 μg, Sigma-Aldrich Co., LLC) was dissolved into dimethyl sulfoxide (DMSO) to 10 μL, then diluted with phosphate-buffered saline to 100 μL (3–15 μM). The cells were then incubated with the probe solution in a 5% CO_2_ atmosphere at 37 °C for 30 min. Afterwards, the supernatant was discarded and the cells were washed twice gently with phosphate-buffered saline, then incubated in McCoy’s 5A (Modified) medium containing 10% FBS for 1 h. When imaging, the supernatant was changed in advance to phenol red-free DMEM (Thermo Fisher Scientific, Inc.).

### Image acquisition

The galvanometer, AOTF, piezo stage, and trigger signals to the EMCCD were all controlled by external voltages. A 32-channel analog-out card (National Instruments, NI PXIe-67s38) provided these voltage signals. It was controlled through a manufacturer-supplied dynamic link library and self-developed software based on LabVIEW. Users can conveniently adjust the voltage range on the galvanometer through the software to change the incident angle below and above the critical angle for a given wavelength. After setting the parameters, the images can be automatically acquired.

Supplementary Figs. 12 and 13 present an example of the control voltages for one frame of the live image sequence shown in Fig. 6. Several features of these voltage signals should be noted, as outlined below:

1. Galvo 1 and 2 comprise Scanning Unit 1 and, control the beam position in one route, while Galvo 3 and 4 comprise Scanning Unit 2 and control the other route. When recording the first half of the image sequence, the TIRF-SIM mode operates, and three pairs of voltages make the orientation of illumination patterns at 0°, 60°, and 120°.
2. We let the piezo stage move forward instead of back and forth to shift the phase to improve the speed of the lateral super-resolution imaging. This is because the push/pull force capacity of the piezo stage is 100/20 N in the operation direction. Moreover, the 12-μm close-loop travel range and 1-nm close-loop resolution are sufficient for the phase shift application.
3. Only Scanning Unit 1 was needed to generate the Ring-TIRF illumination when capturing the second half of the image sequence. Thus, we loaded a relatively high voltage on Galvo 3 and 4 to ensure that the corresponding beam could not enter into the objective. This scheme avoided the mechanical vibration and low switching speed that would have occurred if a shutter was used to control the beam.

### Data processing

Data pre-processing and post-processing were performed with plugins in ImageJ, such as 3D viewer and profile plotting, and by using custom MATLAB codes, such as curve fitting, gradient descent, and alternating direction method of multiplier algorithms. The background leads to misleading depth information and confuses true sample information in all MAIM-like reconstruction algorithms; hence, the background must be subtracted, and interested structures must be segmentted from raw data sets to eliminate the influence of background and improve the analysis efficiency^14,21–24^. Existing segmentation methods are more based on detecting the intensity difference among different pixels. Nonetheless, they are difficult to be used for high-throughput data sets, such as the time-lapse live-cell imaging data and large field of view data, because of different SNR levels in different images and image areas. In this study, we used a machine learning-based segmentation tool to train classifiers and simultaneously and automatically detect and segment image stack with a limited number of input annotations^25^. An example of the possible information loss in the segmentation procedure is shown in Supplementary Fig. 14. Users can also crop images to select several interesting local structures for reconstruction, which are much easier to segment.

### Image display

A color-coded depth representation was adopted to display the 3D super-resolution volume, which is an efficient means of providing axial information content of the sample. The representation was defined by the colorbar based on the multiplication of intensity variations with depth values of each reconstructed image at different depths.

### Code availability

The ADMM code can be found at https://github.com/zcshinee/Pol-TIRF. All other relevant codes are available from the corresponding authors upon reasonable request.

## Electronic supplementary material

Supplementary Information

Description of Additional Supplementary Files

Supplementary Movie 1

Supplementary Movie 2

Supplementary Movie 3

Supplementary Movie 4

## Data Availability

All data are available from the corresponding authors upon reasonable request.

## References

[1] Huang B, Wang W, Bates M, Zhuang X (2008). Three-dimensional super-resolution imaging by stochastic optical reconstruction microscopy. Science.

[2] Pavani SRP (2009). Three-dimensional, single-molecule fluorescence imaging beyond the diffraction limit by using a double-helix point spread function. Proc. Natl Acad. Sci. USA.

[3] Jia S, Vaughan JC, Zhuang X (2014). Isotropic three-dimensional super-resolution imaging with a self-bending point spread function. Nat. Photonics.

[4] Xu K, Babcock HP, Zhuang X (2012). Dual-objective STORM reveals three-dimensional filament organization in the actin cytoskeleton. Nat. Methods.

[5] Aquino D (2011). Two-color nanoscopy of three-dimensional volumes by 4Pi detection of stochastically switched fluorophores. Nat. Methods.

[6] Huang F (2016). Ultra-high resolution 3D imaging of whole cells. Cell.

[7] Bourg N (2015). Direct optical nanoscopy with axially localized detection. Nat. Photonics.

[8] Schmidt R (2008). Spherical nanosized focal spot unravels the interior of cells. Nat. Methods.

[9] Gustafsson MGL (2008). Three-dimensional resolution doubling in wide-field fluorescence microscopy by structured illumination. Biophys. J..

[10] Shao L, Kner P, Rego EH, Gustafsson MG (2011). Super-resolution 3D microscopy of live whole cells using structured illumination. Nat. Methods.

[11] Fiolka R, Shao L, Rego EH, Davidson MW, Gustafsson MGL (2012). Time-lapse two-color 3D imaging of live cells with doubled resolution using structured illumination. Proc. Natl Acad. Sci. USA.

[12] Kraus F (2017). Quantitative 3D structured illumination microscopy of nuclear structures. Nat. Protoc..

[13] Boulanger J (2014). Fast high-resolution 3D total internal reflection fluorescence microscopy by incidence angle scanning and azimuthal averaging. Proc. Natl Acad. Sci. USA.

[14] Stabley DR, Oh T, Simon SM, Mattheyses AL, Salaita K (2015). Real-time fluorescence imaging with 20 nm axial resolution. Nat. Commun..

[15] Cragg GE, So PT (2000). Lateral resolution enhancement with standing evanescent waves. Opt. Lett..

[16] Zheng C (2018). Three-dimensional super-resolved live cell imaging through polarized multi-angle TIRF. Opt. Lett..

[17] Taguchi N, Ishihara N, Jofuku A, Oka T, Mihara K (2007). Mitotic phosphorylation of dynamin-related GTPase Drp1 participates in mitochondrial fission. J. Biol. Chem..

[18] Huang B, Jones SA, Brandenburg B, Zhuang X (2008). Whole-cell 3D STORM reveals interactions between cellular structures with nanometer-scale resolution. Nat. Methods.

[19] Han Y, Li M, Qiu F, Zhang M, Zhang YH (2017). Cell-permeable organic fluorescent probes for live-cell long-term super-resolution imaging reveal lysosome-mitochondrion interactions. Nat. Commun..

[20] Li D (2015). Extended-resolution structured illumination imaging of endocytic and cytoskeletal dynamics. Science.

[21] Winterflood CM, Ruckstuhl T, Verdes D, Seeger S (2010). Nanometer axial resolution by three-dimensional supercritical angle fluorescence microscopy. Phys. Rev. Lett..

[22] Paszek MJ (2012). Scanning Angle Interference Microscopy Reveals Cell Dynamics at the Nano-scale. Nat. Methods.

[23] Chizhik AI, Rother J, Gregor I, Janshoff A, Enderlein J (2014). Metal-induced energy transfer for live cell nanoscopy. Nat. Photonics.

[24] Dos Santos MC, Déturche R, Vézy C, Jaffiol R (2016). Topography of cells revealed by variable-angle total internal reflection fluorescence microscopy. Biophys. J..

[25] Arganda-Carreras I (2017). Trainable Weka Segmentation: a machine learning tool for microscopy pixel classification. Bioinformatics.

