# Supplementary material for: Multi-color live-cell super-resolution volume imaging with multi-angle interference microscopy
Source: Nat Commun. 2018 Nov 16;9:4818. doi: 10.1038/s41467-018-07244-4 (PMC6240104; doi:10.1038/s41467-018-07244-4)
Supplement: Supplementary file 1 — Supplementary Information [file 41467_2018_7244_MOESM1_ESM.pdf]

Multi-color live-cell super-resolution volume imaging with multi-angle interference microscopy

Supplementary Information

Chen & Liu et al.

Supplementary Figure 1: Optical setup of multi-angle interference microscopy (top view)

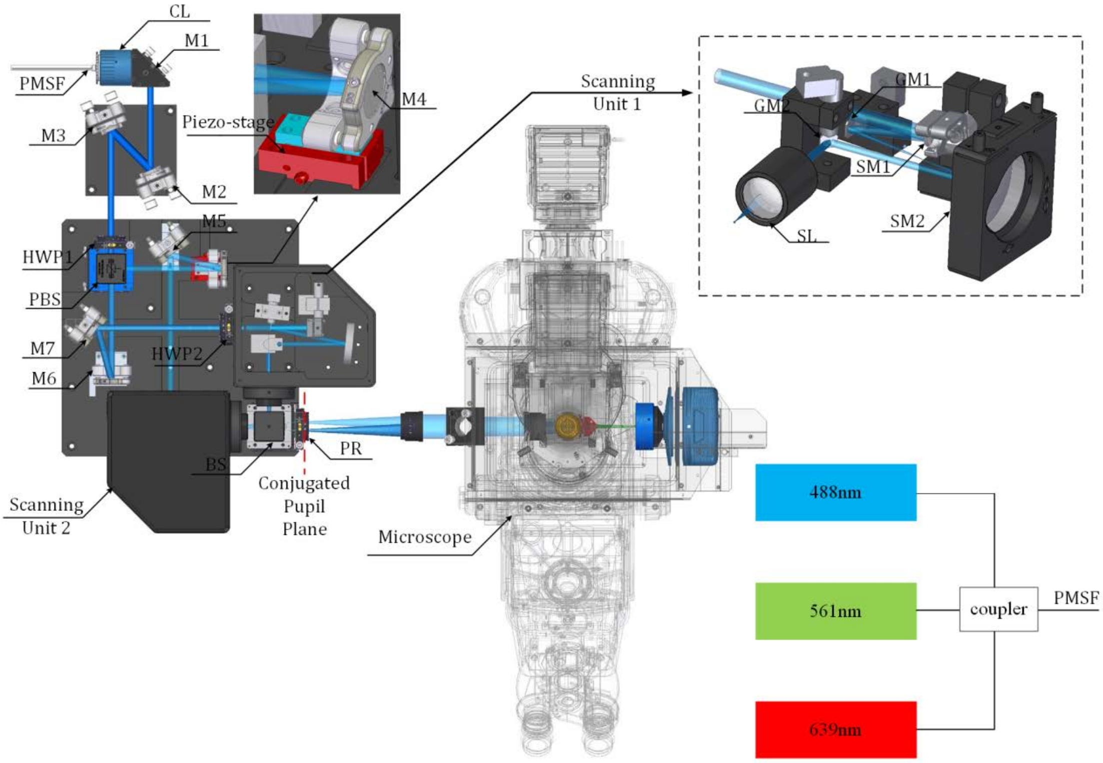

The polarized excitation laser beam (488 nm, 561 nm, and 639 nm) is coupled in the PMSF by a coupler (Nikon LU4A, we herein only provide a schematic at the bottom-right of **Supplementary Fig. 1**) and collimated by the CL. The incident beam can be separated into two beams with the same intensity by the PBS via rotating HWP1. Each scanning unit consists of two galvanometer mirrors (GM1, GM2, 3 kHz scanning speed) and two spherical mirrors (SM1, SM2). The focal lengths of SM1 and SM2 are 100 mm and 50 mm, respectively. The galvanometer and the spherical reflectors form a 4f system, in which SM1 and SM2 are used to project the illumination spot to the position of the rotation axes of the SMY and maintain the spot as fixed at the center of the SMY while the SMX rotates. The chief ray of each beam is parallel to the optical axis again through the SL. The HWP2 before the Scanning Unit2 is applied to maintain the consistency of the polarization direction of the two beams. Through the PR consisting of a liquid crystal cell (LC; Meadowlark, LPR-100-λ) and an achromatic quarter-wave plate (Thorlabs, SAQWP05M-700), all parts of each focused beam are simultaneously converted into s-polarization. After PR, the desired two beams are refocused to the centrosymmetric points near the edge of the BFP of the microscope objective (100×/ 1.49 TIRF, Nikon) by TL1, TL2, and the periscope (**Supplementary Fig. 2**). Each beam is re-collimated by the objective lens and hits the cover slip surface at a tilt angle θ. The illumination angle can be continuously changed through the tip/tilt of the SMX and the SMY in each scanning unit. The phase shift of the illumination pattern is realized by M2 which is fixed on a one-dimensional piezo-platform (PI, P-753).

(PMSF: Polarization maintaining single-mode fiber; CL: Beam collimator; HWP1, HWP2: Half-wave plate; PBS: Polarizing beam splitter cube; BS: Beam splitter cube; M1-M8: Mirrors; SM1, SM2: Spherical

mirrors; GM1, GM2: Scanning galvanometer; SL: Scanning lens; PR: Polarization rotator; TL1-TL3: Tube lens; DM: Dichroic mirror.)

Supplementary Figure 2: Optical setup of multi angle interference microscopy (side view)

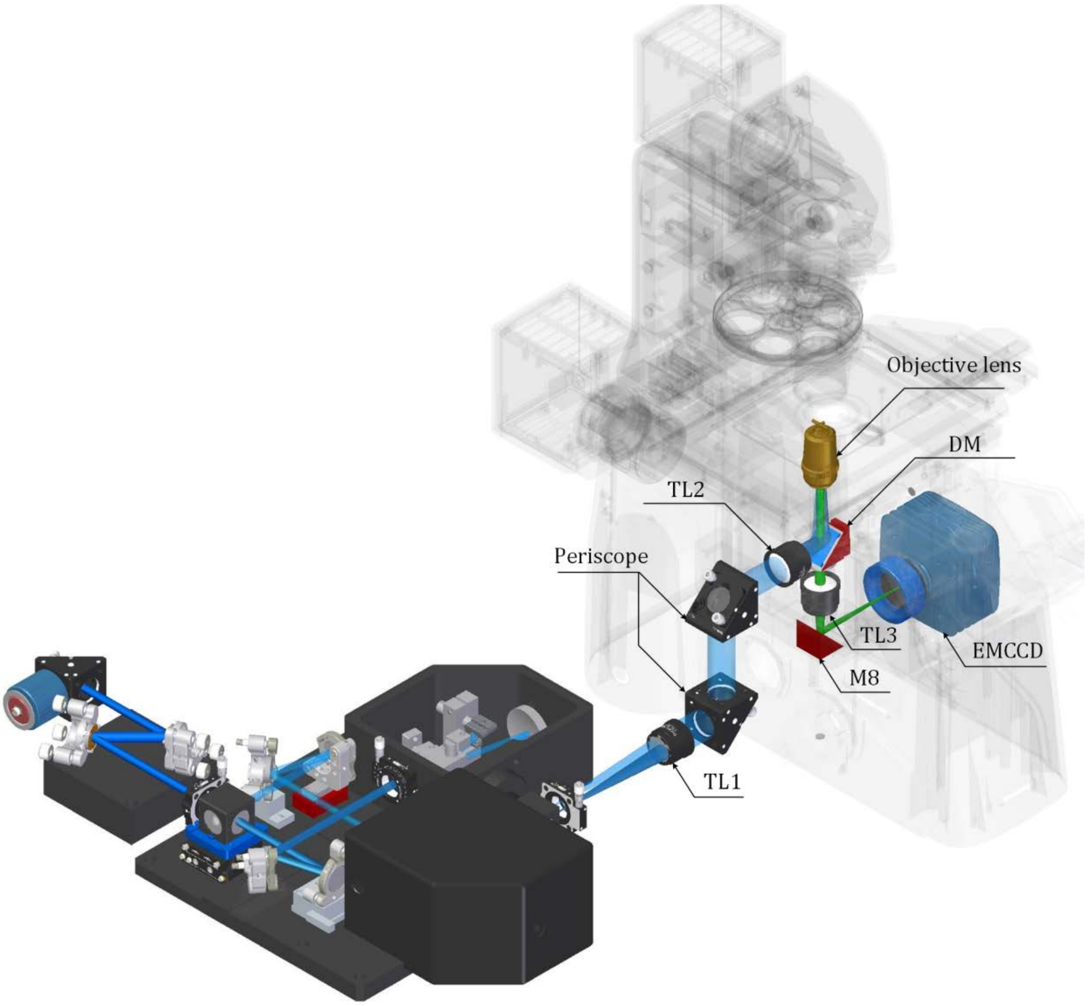

Our system is built on a Nikon-Ti microscope. The figure shows a typical setup of the system combined with the microscope (not exactly consistent with the actual setting). Each beam is recollimated by the objective (100×/1.49 TIRF, Nikon) and hits the cover slip surface at an angle that exceeds the critical angle for the total internal reflection. Evanescent waves from the two beams extend into the specimen and interfere there to produce a line excitation pattern. The penetration depth can be adjusted by continuously changing the illumination angle. The emission fluorescence light (shown as green) from the specimen is collected by the camera (EMCCD, Andor, iXon Ultra 888) after the DM. Most of the CAD models of the optical elements used in **Supplementary Fig. 1** and **2** were derived from Thorlabs and Newport websites. Permission to use granted by Thorlabs Corporation. All rights reserved. Permission to use granted by Newport Corporation. All rights reserved.

Supplementary Figure 3: Diagram and theory of 3D super-resolution reconstruction

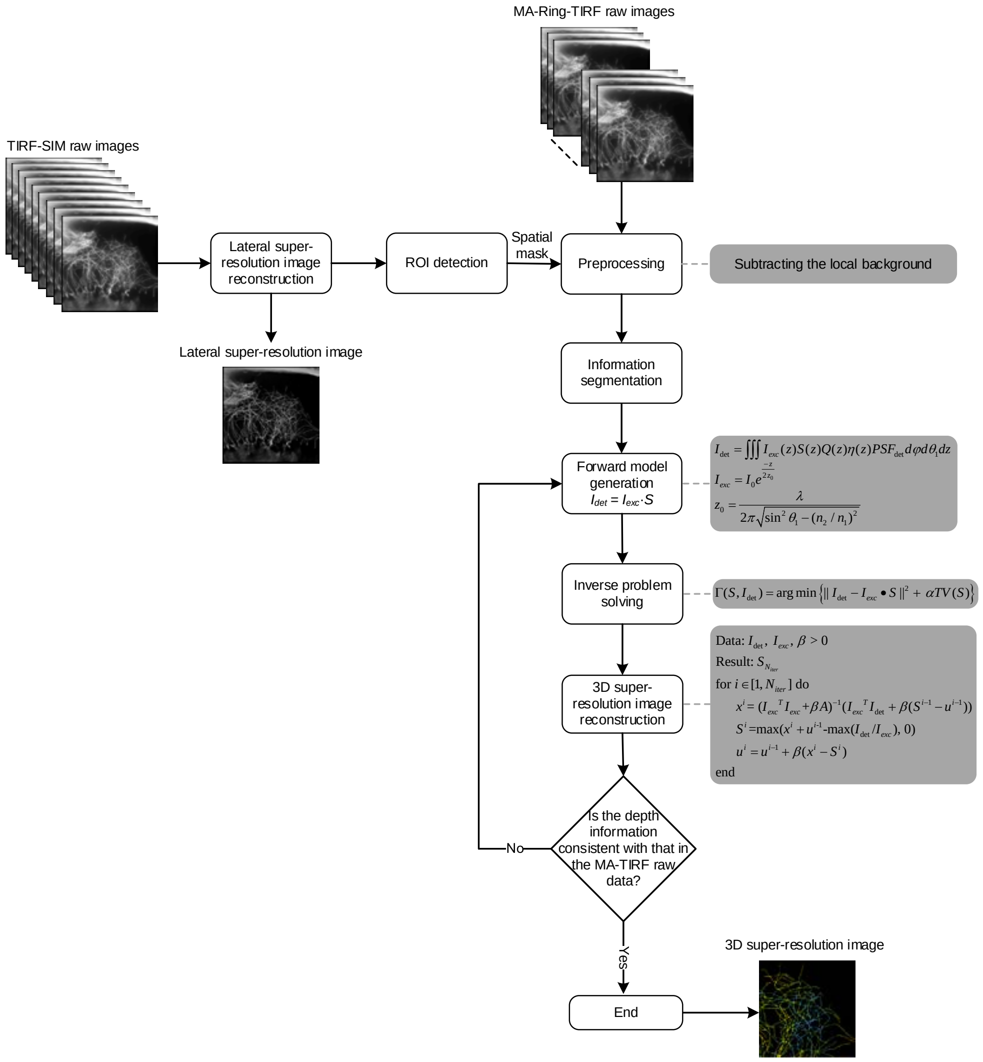

A real-time 3D super-resolution reconstruction is performed using home-made software written in MATLAB. The acquired raw data includes the TIRF-SIM and MA-Ring-TIRF raw images. The TIRF-SIM raw images should be performed at a low incident angle to provide lateral super resolution projected over the whole axial range that MA-TIRF then covers. After that, a 2D super-resolution reconstruction is performed by extracting the high-frequency information of the TIRF-SIM raw images and down-shifting them. On the latter, the lateral super-resolution image is thresholded then regarded as a binary spatial mask for subtracting background and segmenting and extracting more detailed lateral information from the acquired MA-Ring-TIRF raw image stacks, thereby abandoning the diffraction-limited information. We may detect the same ROI for all images to improve the reconstruction efficiency. The resulting segmentation images are served as the input of the following 3D reconstruction.

For reconstructing the 3D super-resolution image, the forward model is generated as follows. The acquired fluorescence signal in each single-angle TIRF image can be written as

$$I_{\det} = \iiint I_{exc}(z)S(z)Q(z)\eta(z)PSF_{\det} d\varphi d\theta dz \quad (1)$$

where $I_{exc}(z)$ denotes the excitation field of the evanescent wave, $S(z)$ denotes the local distribution of the dye molecules in the sample, and $Q(z)$ represents the parameters that affect the fluorescence excitation, such as the scattering, quantum efficiency, absorption rate, dipole orientation, and decay rate of the fluorescent dyes. In addition, $\eta(z)$ denotes the detection efficiency, $PSF_{\det}$ represents the detection PSF of the system, and φ , θ and z respectively denote the incident angle, azimuthal angle and depth of the sample.

Note that the image value $I_{\det}$ is not only managed by the evanescent field, but also particularly affected by $Q(z)$, $\eta(z)$, and $PSF_{\det}$ which is too difficult to determine for quantitatively analyze the TIRF image. Fortunately, these parameters mentioned above depend mainly on the depth z of the molecules; they do not relate to the incident angle θ. In other words, their values remain constant when changing the incident angle. Thus, by averaging the images acquired at different incident angles, we can eliminate the influence of these unknown parameters, which is also the theoretical basis of multi-angle evanescent wave illumination.

The excitation field of evanescent wave can be written as

$$I_{exc} = I_0 e^{\frac{-z}{2z_0}} \quad (2)$$

where I_0 is the intensity at the interface given by Fresnel’s equation under s-polarization, and z_0 is the penetration depth expressed as

$$z_0 = \frac{\lambda}{2\pi\sqrt{\sin^2\theta - (n_2 / n_1)^2}} \quad (3)$$

where λ is the wavelength of the incident light, and n_2 and n_1 are the refractive index of the incident medium and refracted medium, respectively.

Considering that the amounts of the incident angle and reconstruction plane are finite, Eq. (1) can be discretized into a matrix form

$$I_{\det} = I_{exc} \cdot S \quad (4)$$

where $I_{\det}$ is a matrix of size $N_{angles} \times N_{pixelx} \times N_{pixely}$ representing the obtained stack of images, I_{exc} denotes a matrix of size $N_{angles} \times N_{planes}$ representing the intensity distribution of the evanescent wave at different incident angles, and S is the matrix of size $N_{planes} \times N_{pixelx} \times N_{pixely}$ representing the 3D distribution of the sample. N_{angles} , N_{pixelx} , N_{pixely} and N_{planes} are the incident angle number, pixel number in the x direction in each 2D image, pixel number in the y direction in each 2D image, and the reconstructed plane number, respectively.

As a result, the reconstruction of the 3D sample distribution S turns into solving the convex optimization problem, namely, finding the optimal S to minimize the following objective function

$$\Gamma(S, I_{\det}) = \arg\min\left\{\| I_{\det} - I_{exc} \cdot S \|^2 + \alpha TV(S)\right\} \tag{5}$$

where $TV(S)$ is the regularization term that is added so that the solution of the pathological problem is close as possible to the optimal solution in the actual situation and α is the limiting factor balancing the terms.

There are many kinds of algorithms to solve the problem, such as the inverse Laplace transform[1], Bayesian framework[2], Lawson and Hanson (NNLS) algorithm[3], and parallel proximal algorithm (PPXA)[3]. However, each of these methods has its weaknesses. The Laplace transform is not appropriate for reconstructing the sample with continuous structures, such as microtubules, the Bayesian framework requires obtaining the shape prior information of biological structures, NNLS is susceptible to the imaging noise, and PPXA is limited to the reconstruction speed.

With the experimental results, we utilized our previously reported MA-TIRF algorithm for a fast reconstruction[4]. The corresponding code has been open-sourced in GitHub repository[5]. This algorithm, based on the alternating direction method of multipliers (ADMM), is faster and more robust in many applications because it decomposes the optimization problem into several sub-problems to solve (**Supplementary Fig. 7**). The corresponding flow diagram of the algorithm is given.

Data: $I_{\det}$, I_{exc}, $\beta > 0$
Result: $S_{N_{iter}}$
for $i \in [1, N_{iter}]$ do
  $x^i = (I_{exc}{}^T I_{exc} + \beta A)^{-1}(I_{exc}{}^T I_{\det} + \beta(S^{i-1} - u^{i-1}))$
  $S^i = \max(x^i + u^{i-1} - \max(I_{\det}/I_{exc}), 0)$
  $u^i = u^{i-1} + \beta(x^i - S^i)$
end

By minimizing the objective function, the 3D super-resolution image is obtained. Finally, our software compares the axial distribution in the MA-TIRF images and super-resolution images to confirm the validity of the reconstruction. It should be noted that the curve fitting method can also reconstruct the depth information of the sample; however, it cannot obtain the images at different depths and thus cannot achieve 3D visualization[6].

Supplementary Figure 4: Optical sectioning capacity of lateral super-resolution reconstruction

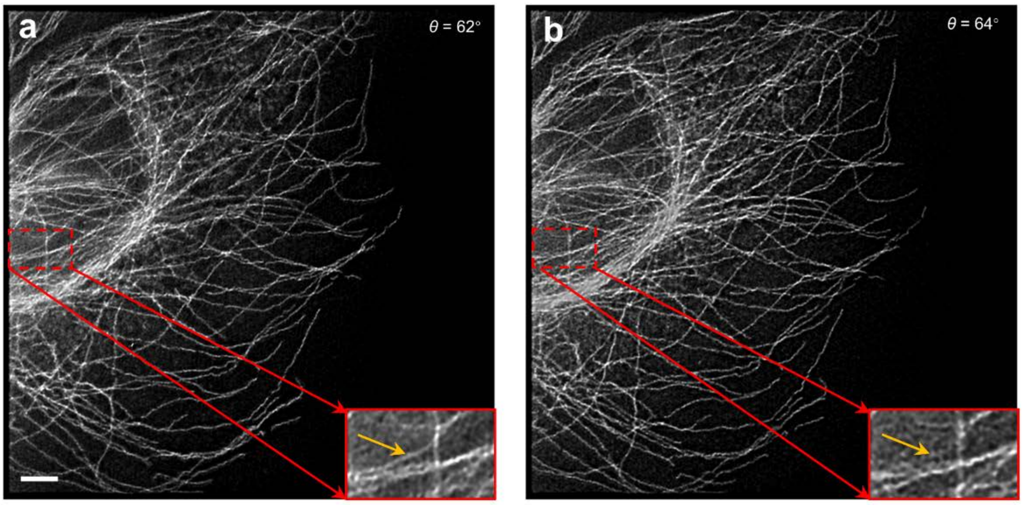

In order to demonstrate the optical sectioning capacity of the lateral super-resolution reconstruction, we obtained the TIRF-SIM images at different incident angles of (a) θ = 62° and (b) θ = 64°. Different parts of the sample were illuminated and excited because of the different penetration depths of the evanescent waves at different incident angles. As shown in the magnified image of the boxed region (red), one microtubule in particular is visible and invisible at θ = 62° and θ = 64°, respectively (arrow). Scale bar, 5 μm.

Supplementary Figure 5: Influence of azimuthal averaging on TIRF imaging

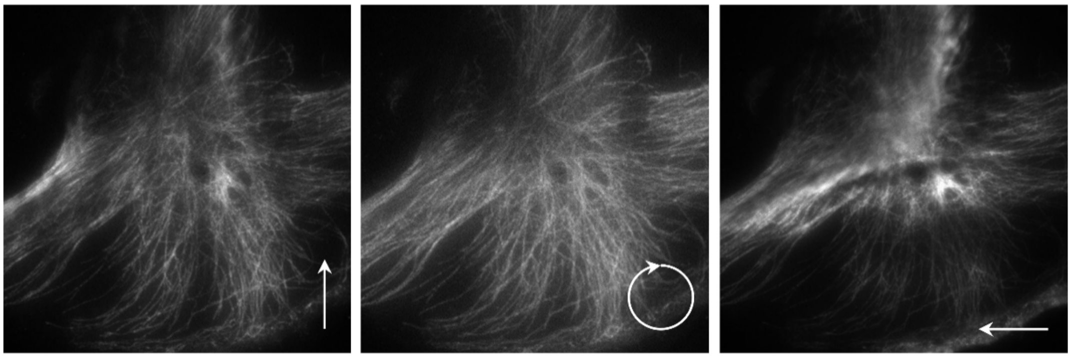

Comparison of single-spot TIRF images at different illumination directions (left, right) and the Ring-TIRF image by azimuthal averaging (middle) recorded with the same sample of microtubules in U373 human astrocytes. The arrowheads represent the illumination directions. Because of the large-angle oblique illumination characteristic of the single-spot TIRF, the intensity distribution of an image obtained under a single azimuthal angle is uneven and asymmetric. Fringes exist along the illumination directions, which may cause artifacts when quantitatively analyzing TIRF images and reconstructing the depth. In contrast, the Ring-TIRF image is homogeneously illuminated.

Supplementary Figure 6: Theoretical verification of MAIM

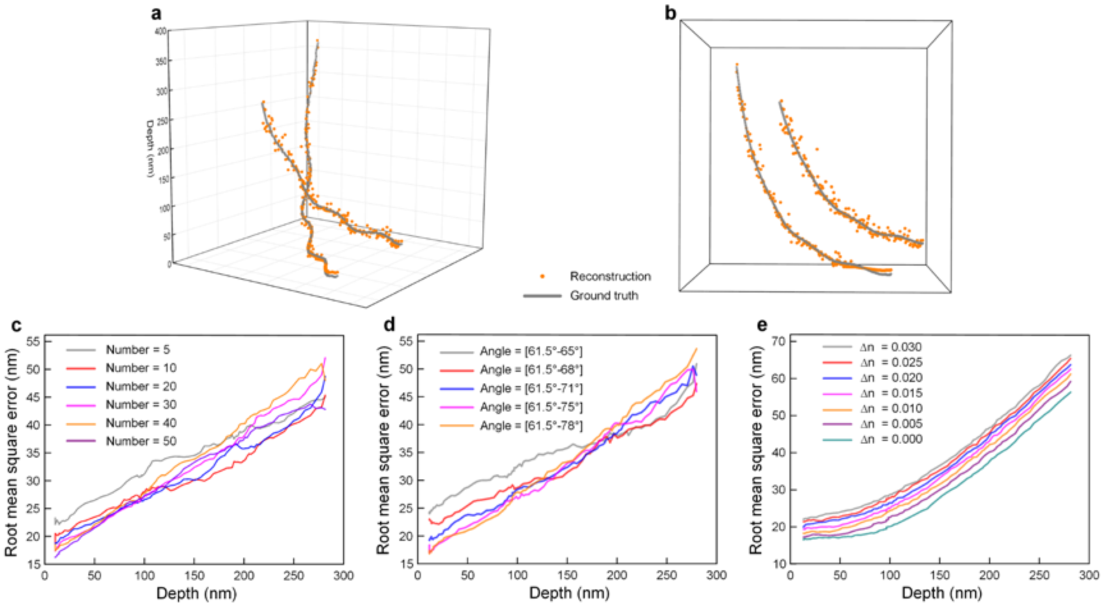

(**a**) Front view and (**b**) side view of the ground truth sample and the reconstruction result. The simulation conditions were: λ = 488 nm, n_1 = 1.515, n_2 = 1.33, SNR = 30, θ = [61.5°–71°], and angle number = 20. Root mean square error of the calculated depth from the true position under (**c**) different angle numbers, (**d**) different angle varying ranges, and (**e**) different refractive indices n_2. In (**e**), Δn is the difference of the refractive index n_2 used to generate matrices I_{exc} and $I_{\det}$ in Supplementary Equation 4.

Supplementary Figure 7: Experimental verification of MAIM

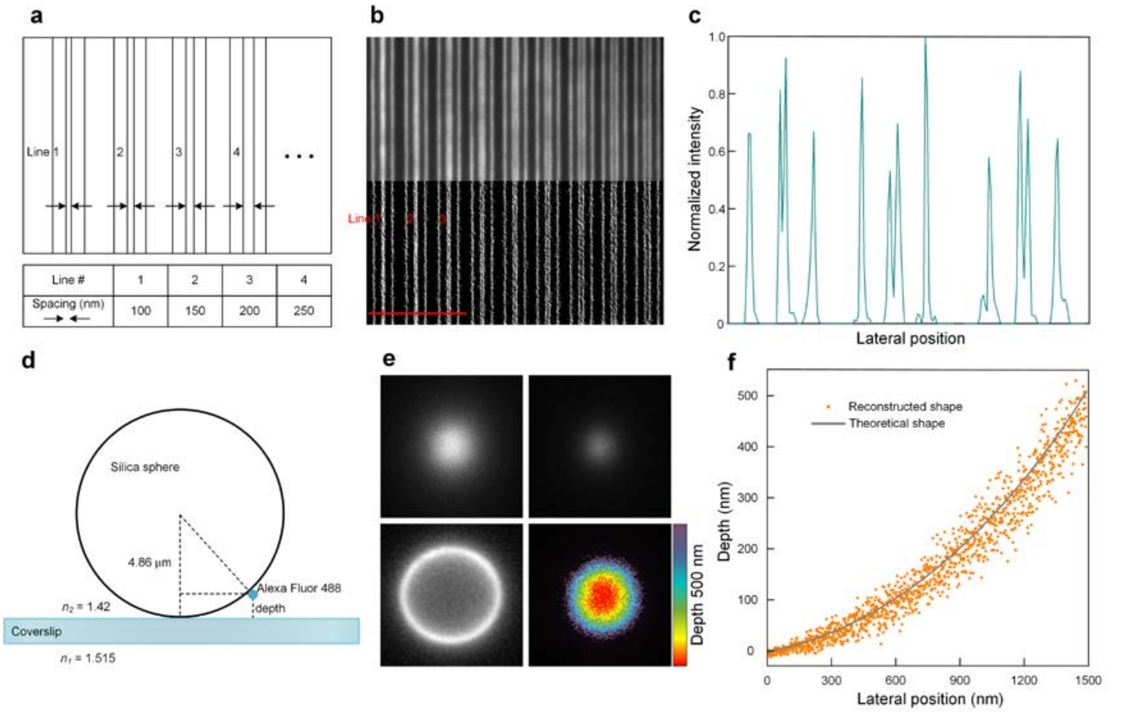

(**a**–**c**) Experimental verification of the lateral resolution enhancement with gradually spaced lines ground-truth sample (Argo-HM, 488 nm excitation). (**a**) Schematics of the gradually spaced lines ground-truth sample. (**b**) Experimental comparison of the diffraction-limited wide-field image (top) and SIM image (bottom). (**c**) Intensity profile along the red line in (**b**). (**d**–**f**) Experimental verification of the axial resolution enhancement with 5-μm silica sphere ground-truth sample (Bangs Laboratories, Inc.). (**d**) Schematics of a 4.86 ± 0.47 μm silica sphere labeled with Alexa Fluor 488 deposited on a coverslip. (**e**) Experimental comparison of the TIRF images acquired at θ = 69.75° (top left) and θ = 72° (top right), wide-field image (bottom left), and reconstructed axial super-resolution image (bottom right). The experimental conditions were: λ = 488 nm, n_1 = 1.515, n_2 = 1.42, θ = [69.75°-74°], and angle number = 20. (**f**) Theoretical (gray line, diameter = 4.86 μm) and measured (orange dots, diameter = 4.98 μm) depth profiles from the center of the sphere. The measurements were performed for 20 beads.

Supplementary Figure 8: Comparison of reconstructed results with different algorithms

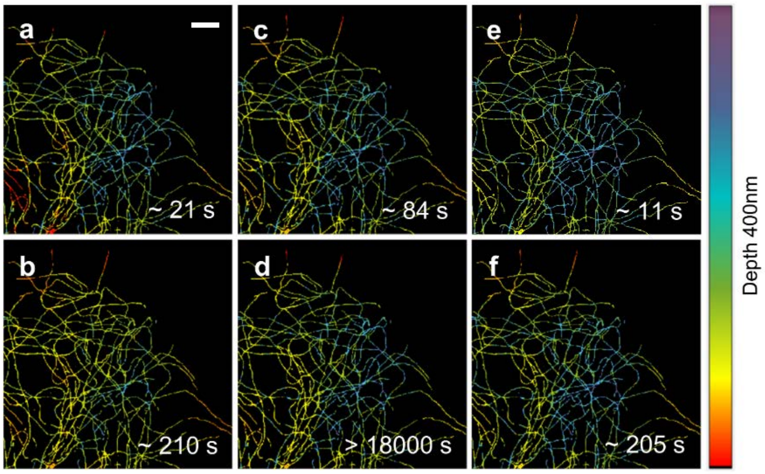

The same sample shown in **Fig. 1a–c** reconstructed with different algorithms. (**a**) Gradient descent algorithm (GD), (**b**) gradient descent with total-variation regularization algorithm (GD_TV), (**c**) PPXA algorithm, (**d**) PPXA with total-variation regularization algorithm (PPXA_TV), (**e**) ADMM algorithm, and (**f**) ADMM with total-variation regularization algorithm (ADMM_TV). The time consumed for reconstruction with different algorithms of the same 561×561 image sizes and 20 iterations is listed at the bottom-right of each figure. ADMM is the optimal choice for reconstruction. Scale bar, 3 μm.

Supplementary Figure 9: Comparison of MA-TIRF and MAIM

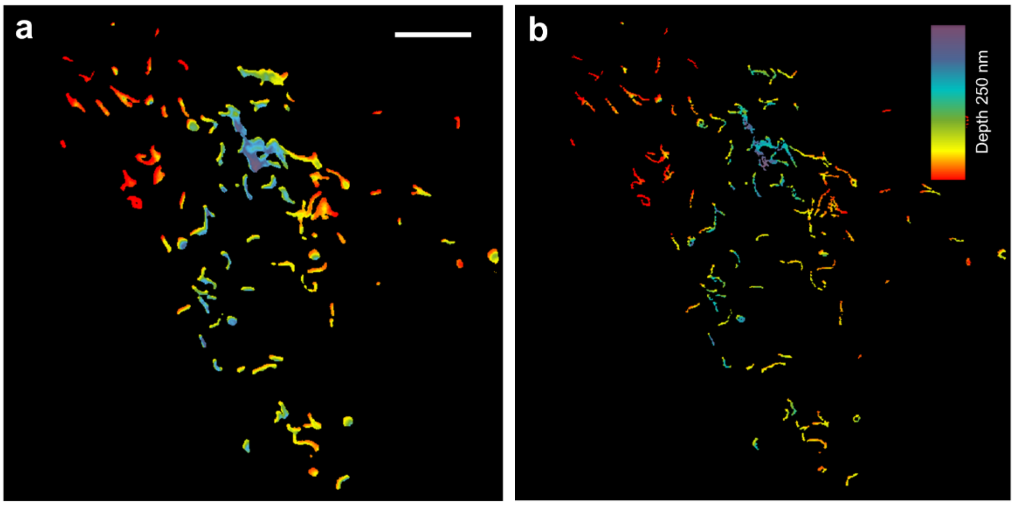

(**a**) MA-TIRF reconstruction image and (**b**) MAIM reconstruction image of STAR 580-labeled peroxisomes shown in **Fig. 1d–f**. The MA-TIRF image was reconstructed using the method presented in Ref. 13, which has the same depth distribution but a diffraction-limited lateral resolution compared to the color-coded image reconstructed with our method. Scale bar, 3 μm.

Supplementary Figure 10: 3D reconstruction results

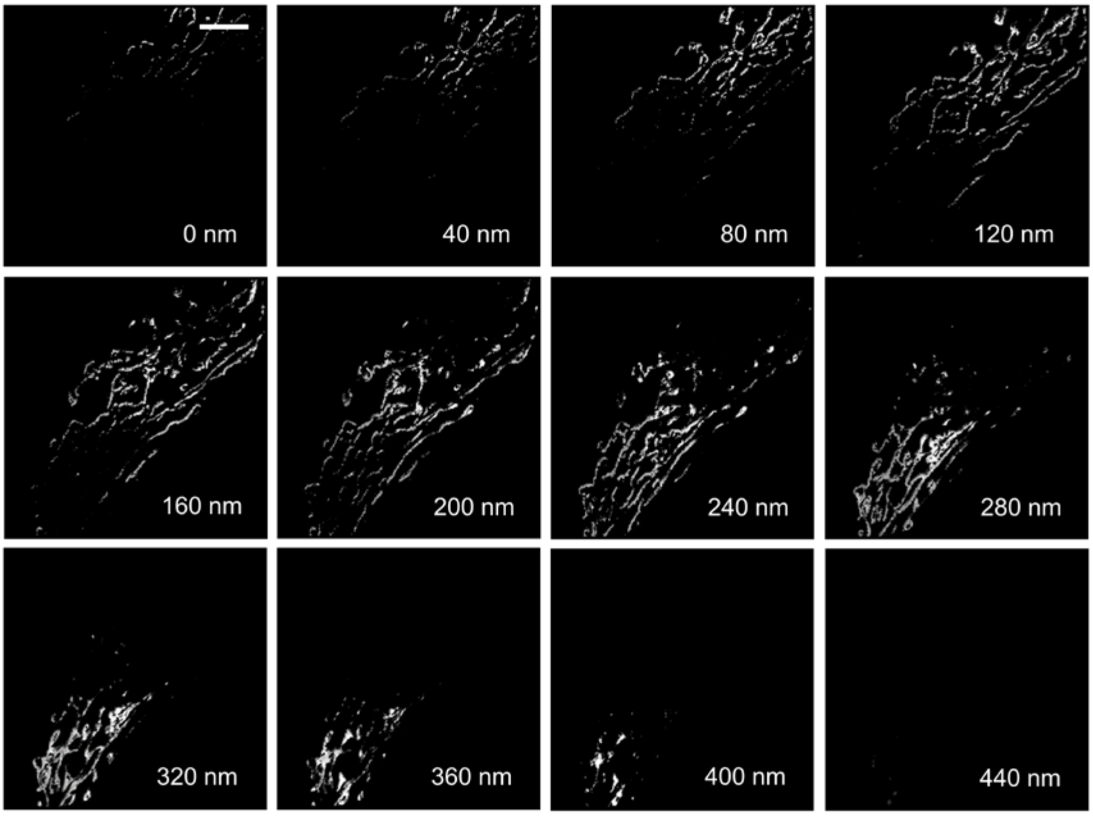

Absolute depth and optical sectioning of the mitochondria sample shown in **Fig. 2** after 3D reconstruction. Their continuity and the morphology change from the surface (0 nm) to the deepest plane (440 nm) are shown. The depth-resolved ability for different samples depends on the acquisition parameters, such as sample depth, number of incident angles, and number of reconstructed image planes. Scale bar, 5 μm.

Supplementary Figure 11: Calibration of the relationship between the voltage of the galvanometer and the angle of incidence

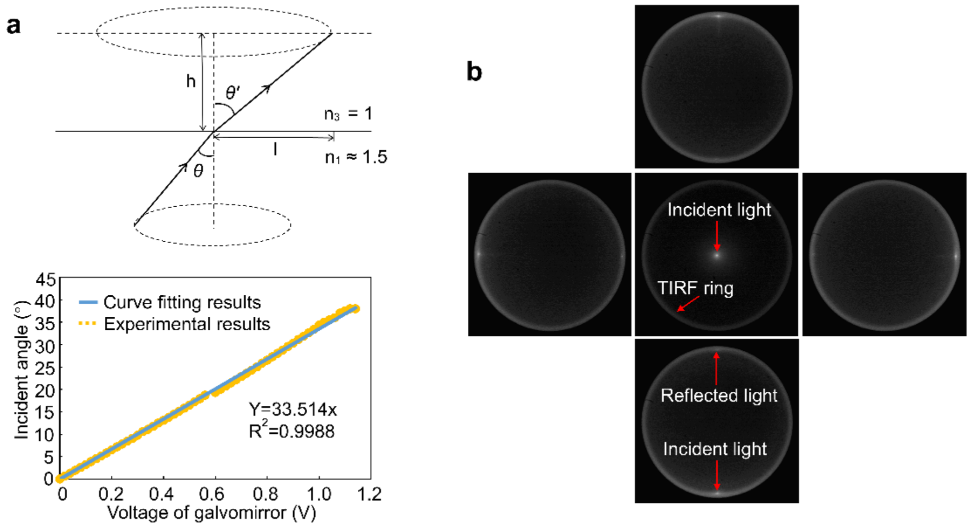

(**a**) The reflected angle θ' from the objective is changed by varying the voltages of the galvanometer continuously and metered according to the position of the refracted parallel light spot along the laboratory roof. The corresponding incident angle is calculated from Snell's law. The relationship between the galvanometer voltage and the angle of incidence is determined by the geometrical relationship. This method shows a linear relationship between the voltage of the galvanometer and the angle of incidence with a slope of 33.514 and a degree of fitting of 0.9988.

(**b**) Experimental images of the BFP of the objective at a critical angle for different azimuthal angles recorded with the sample of a monolayer of fluorescent particles (F8803) fixed on the glass/water interface. Middle: voltage = 0 V; right: voltage = 1.831 V, α = 0°; top: voltage = –1.833 V, α = 90°; left: voltage = –1.832 V, α = 180°; and bottom: voltage = 1.832 V, α = 270°. According to the fitting relationship from (**a**), the voltage of the galvanometer corresponding to the critical angle of the same particle sample is 1.83 V, which is slightly different from the experiment value of 1.832 V. The error is caused by the limitation of the geometric measurement itself. Through the procedure of BFP imaging, we can correct the slope to 33.5. In addition, the asymmetry of the voltages of the galvanometer for different azimuthal angles can be compensated by elliptical scanning. Note that because this linear calibration relationship was obtained from the geometrical relationship and Snell's law which is irrelevant with the refractive index of the sample, it can be applied to any interfaces and will not affect the reconstruction[7].

Supplementary Figure 12: Example of control voltages for one frame of a live image sequence.

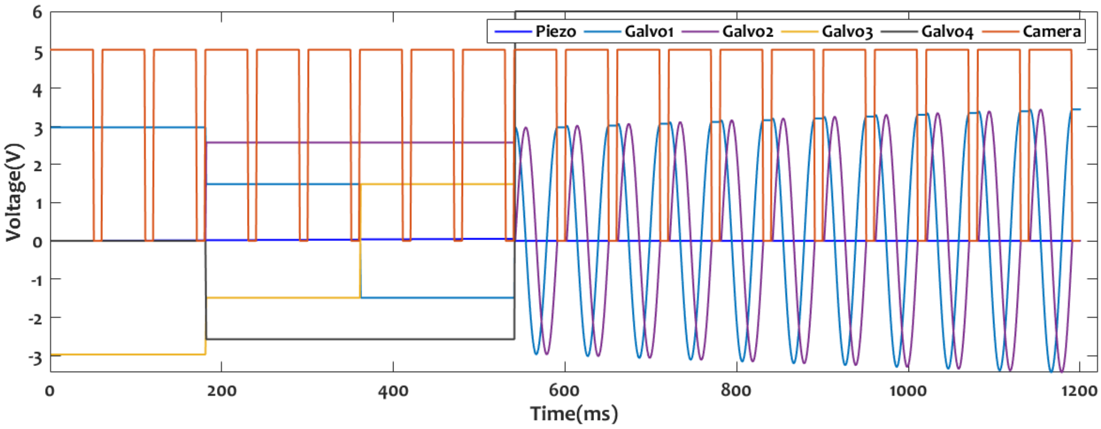

See **Online Methods** for the accompanying description.

Supplementary Figure 13: Control scheme for rapid piezo stage movement for one frame of live image sequence.

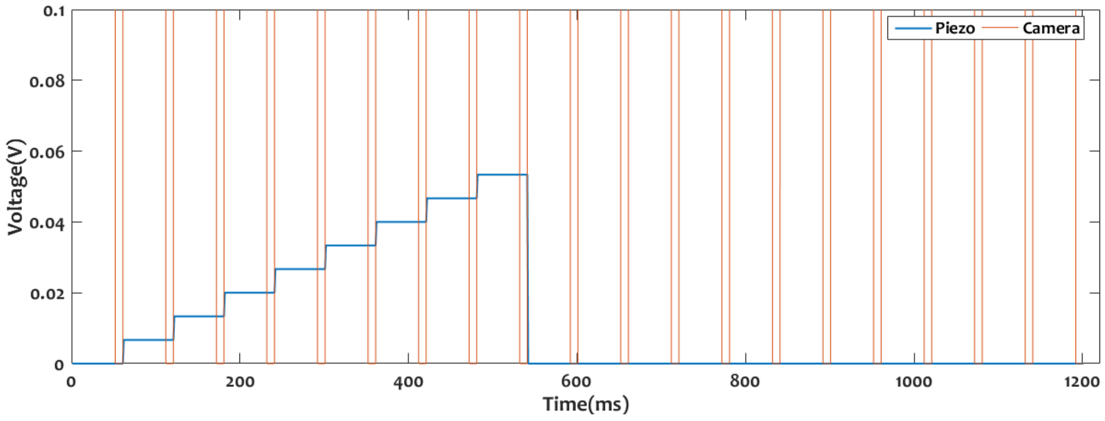

Higher magnification view of the voltages in **Supplementary Fig. 11**, illustrating a step voltage routine that enables <5 ms piezo movement with minimal vibration and time. See **Online Methods** for further details.

Supplementary Figure 14: An example of the possible information loss in the MAIM reconstruction

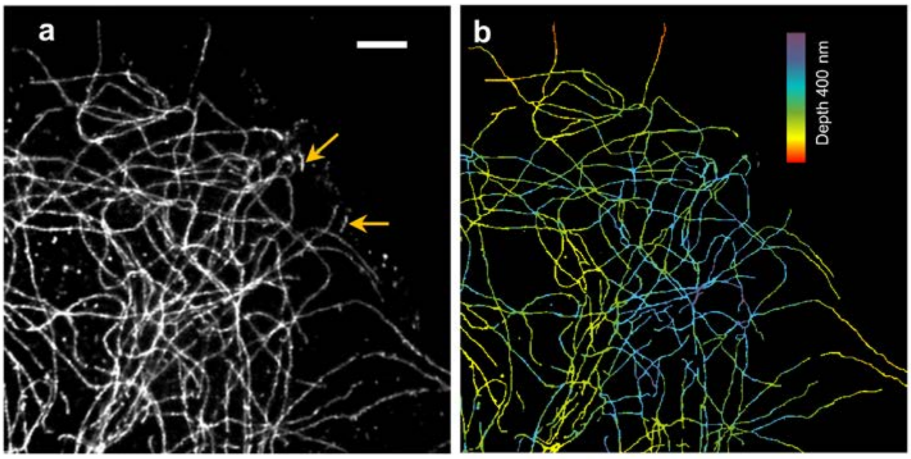

(a) TIRF-SIM image and (b) incomplete MAIM reconstruction image of microtubules labeled with Alexa Fluor 488 ($\lambda = 488$ nm) in U373 cells shown in **Fig. 1b** and **c**. Several sample structures were lost (orange arrowheads) after MAIM reconstruction, which is caused by the inadequate information segmentation. Scale bar, 3 µm.

Supplementary Note 1: Angle calibration

The penetration depth of the evanescent wave determined by the incident angle of the illumination light, must be precisely calculated to quantitatively analyze MAIM images. In our current optical system, the incident angle is controlled by galvanometer scanning under different voltages. Thus, the calibration problem becomes determining the relationship between the galvanometer voltage and the angle of incidence. When the light propagates from the objective into the air, the refracted parallel light is a spot on the laboratory roof. The angle of reflection θ' can be calculated from the geometrical correlation

$$\theta' = \arctan(\tfrac{l}{h}) \qquad (1)$$

where l is the lateral distance of the light spot relative to the middle of the objective, and h is the height of the roof.

The incident angle θ can then be deduced from Snell's law,

$$\theta = \arcsin(\frac{n_3}{n_1}\sin\theta') \qquad (2)$$

where n_3 and n_1 are the refractive index of the air and objective, respectively.

By continuously changing the voltages of the galvanometer, a series of angles and voltages are obtained, which could then be fitted to determine their relationship. Accordingly, we found a linear correlation between the voltage of the galvanometer and the angle of incidence with a slope of 33.514 and a degree of fitting of 0.9988 (**Supplementary Fig. 11**).

To further improve the calibration precision, we imaged the BFP of the objective at different voltages of the galvanometer and different azimuthal angles with the sample of a monolayer of fluorescent particles fixed on the glass/water interface (**Supplementary Fig. 11**, **Supplementary Movie 4**). After this procedure, we could correct the slope to 33.5. The calibration was performed over all galvanometer voltages. This linear calibration relationship between the incident angle and the galvanometer voltage is only related to the optical system and suitable for any interface experiment.

The refractive index of different biological samples may be different, which affects the reconstruction (**Supplementary Fig. 6e**); hence, we need to precisely determine the refractive index of a sample in each experiment to reduce the reconstruction error. To achieve this, we first find the "critical voltage" of the experimental sample by imaging the objective BFP. With the "critical voltage", the critical angle of this special sample can be easily calculated from the initial calibration relationship. Finally, the effective refractive index of this sample can be obtained, then used for the MAIM reconstruction that follows. Note that the small index difference between different regions of the same sample was ignored in the reconstruction[8,9].

Supplementary Note 2: Factors affecting 3D reconstruction

According to our simulations and experiments, the imaging performance of our approach is mainly governed by the calibration of the critical angle and the SNR, especially in live-cell imaging.

(1) Angle calibration

The 3D reconstruction of the sample requires first building the forward model between the incident evanescent wave field and the acquired image stacks. The distribution of the evanescent field is determined by the incident angle, which is further controlled by the galvanometer voltage. Therefore, the precise calibration of the relationship between the voltage and the angle will directly affect the reconstruction quality (**Supplementary Fig. 11**, **Supplementary Note 1**).

(2) SNR

The SNR is also of great importance when reconstructing a high-quality 3D super-resolution image because of the following reasons: (i) in lateral super-resolution reconstruction, the high-frequency information generated by multiple beam interference must be shifted to their correct position. However, the higher the frequency, the lower the SNR and the more difficult the reconstruction as a result of the low-pass-filter characteristic of the imaging system; (ii) the noise and background in the image stacks may lead to incorrect depth information and confuse the true sample information, and thus need to be subtracted[3, 6, 9, 10]; (iii) the image thresholding and information segmentation procedures in the MAIM reconstruction will be easier and more accurate as the SNR increases[6]; and (iv) the higher the SNR, the higher the 3D resolution (**Fig. 2**).

(3) Axial overlapping

One potential limitation of the MAIM reconstruction is that when an object larger than the diffraction limit is located in the same lateral position, but different axial positions, as a smaller object, the smaller object may be axially resolved in the 3D reconstruction; however, the smaller object will no longer exhibit super-resolution in the lateral dimension becasue the TIRF-SIM mask will have the lateral dimension of the larger object. In contrast, a more common case is that the sizes of possible axial overlapping structures are similar and less than the diffraction limit, which means that they should appear to be the same size in the image. Under this circumstance, the effect of the TIRF-SIM generated mask on the lateral resolution could probably be neglected. Nevertheless, users should be aware of this potential artifact.

Supplementary References

1 Olveczky, B. P., Periasamy, N. & Verkman, A. S. Mapping fluorophore distributions in three dimensions by quantitative multiple angle-total internal reflection fluorescence microscopy. *Biophys. J.* **73**, 2836-2847 (1997).

2 Yang, Q., Karpikov, A., Toomre, D. & Duncan, J. S. 3-D reconstruction of microtubules from multi-angle total internal reflection fluorescence microscopy using Bayesian framework. *IEEE Trans. Image Process.* **20**, 2248-2259 (2011).

3 Boulanger, J. *et al.* Fast high-resolution 3D total internal reflection fluorescence microscopy by incidence angle scanning and azimuthal averaging. *Proc. Natl. Acad. Sci. USA* **111**, 17164-17169 (2014).

4 Zheng C. *et al.* Three-dimensional super-resolved live cell imaging through polarized multi-angle TIRF. *Opt. Lett.* **43**, 1423-1426 (2018).

5 GitHub [https://github.com/zcshinee/Pol-TIRF].

6 Dos Santos, M. C., Déturche, R., Vézy, C. & Jaffiol, R. Topography of cells revealed by variable-angle total internal reflection fluorescence microscopy. *Biophys.J.* **111**, 1316-1327 (2016).

7 Paszek, M. J. *et al.* Scanning Angle Interference Microscopy Reveals Cell Dynamics at the Nano-scale. *Nat. Methods* **9**, 825-827 (2012).

8 Dos Santos, M. C., Déturche, R., Vézy, C. *et al.* Axial nanoscale localization by normalized total internal reflection fluorescence microscopy. *Opt. Lett.* **4**, 869-872 (2014).

9 Stabley, D. R., Oh, T., Simon, S. M., Mattheyses, A. L. & Salaita, K. Real-time fluorescence imaging with 20 nm axial resolution. *Nat. Commun.* **6**, 8307 (2015).

10 Bourg, N. *et al.* Direct optical nanoscopy with axially localized detection. *Nat. Photonics* **9**, 587-593 (2015).
