# Supplementary material for: Multi-color live-cell super-resolution volume imaging with multi-angle interference microscopy
Source: Nat Commun. 2018 Nov 16;9:4818. doi: 10.1038/s41467-018-07244-4 (PMC6240104; doi:10.1038/s41467-018-07244-4)
Supplement: Supplementary file 2 — Description of Additional Supplementary Files [file 41467_2018_7244_MOESM2_ESM.pdf]

Description of Additional Supplementary Files

File Name: Supplementary Movie 1
Description: MAIM imaging of 3D rotational morphology change of fixed mitochondria labeled with MitoTracker® Red CMXRos in a bovine pulmonary artery endothelial cell. The same field of view is shown in Fig. 4.

File Name: Supplementary Movie 2
Description: MIAM imaging of 3D dynamics of mitochondria fusion and fission labeled with Atto 647N in a live U2OS cell at time intervals of 2s. The same field of view is shown in Fig. 6a, b.

File Name: Supplementary Movie 3
Description: MIAM imaging of 3D dynamics of mitochondria fusion and fission labeled with Atto 647N in a live U2OS cell at time intervals of 2s (3D visualization). The same field of view is shown in Fig. 6a, b.

File Name: Supplementary Movie 4
Description: Imaging objective BFP at different incident angles and azimuthal angles using the sample of a monolayer of fluorescent particles fixed on the glass/water interface.
